# A Spatially Explicit Model of Functional Connectivity for the Endangered Przewalski’s Gazelle (*Procapra przewalskii*) in a Patchy Landscape

**DOI:** 10.1371/journal.pone.0080065

**Published:** 2013-11-08

**Authors:** Chunlin Li, Zhigang Jiang, Hongxia Fang, Chunwang Li

**Affiliations:** 1 School of Resources and Environmental Engineering, Anhui University, Hefei, Anhui, China; 2 Key Laboratory of Animal Ecology and Conservation Biology, Institute of Zoology, Chinese Academy of Sciences, Beijing, China; University of Waikato (National Institute of Water and Atmospheric Research), New Zealand

## Abstract

**Background:**

Habitat fragmentation, associated with human population expansion, impedes dispersal, reduces gene flow and aggravates inbreeding in species on the brink of extinction. Both scientific and conservation communities increasingly realize that maintaining and restoring landscape connectivity is of vital importance in biodiversity conservation. Prior to any conservation initiatives, it is helpful to present conservation practitioners with a spatially explicit model of functional connectivity for the target species or landscape.

**Methodology/Principal Findings:**

Using Przewalski’s gazelle (*Procapra przewalskii*) as a model of endangered ungulate species in highly fragmented landscape, we present a model providing spatially explicit information to inform the long-term preservation of well-connected metapopulations. We employed a Geographic Information System (GIS) and expert-literature method to create a habitat suitability map, to identify potential habitats and to delineate a functional connectivity network (least-cost movement corridors and paths) for the gazelle. Results indicated that there were limited suitable habitats for the gazelle, mainly found to the north and northwest of the Qinghai Lake where four of five potential habitat patches were identified. Fifteen pairs of least-cost corridors and paths were mapped connecting eleven extant populations and two neighboring potential patches. The least-cost paths ranged from 0.2 km to 26.8 km in length (averaging 12.4 km) and were all longer than corresponding Euclidean distances.

**Conclusions/Significance:**

The model outputs were validated and supported by the latest findings in landscape genetics of the species, and may provide impetus for connectivity conservation programs. Dispersal barriers were examined and appropriate mitigation strategies were suggested. This study provides conservation practitioners with thorough and visualized information to reserve the landscape connectivity for Przewalski’s gazelle. In a general sense, we proposed a heuristic framework for species with similar biological and ecological characteristics.

## Introduction

Habitat fragmentation, associated with human population expansion and the following landscape modification, is of major concern to agencies of wildlife conservation [1,2]. Disconnected landscape impedes dispersal, reduces gene flow and aggravates inbreeding in endangered species [3]. Well-connected landscape is considered necessary for long-term viability of extinction-prone metapopulation [2,4]. Since Merriam [5] firstly introduced the concept and Taylor et al. [6] defined it as “the degree to which the landscape impedes or facilitates movement among resource patches”, people are putting increasing efforts in maintaining and restoring landscape connectivity.

Numerous connectivity models have been put forward but there has been no consensus over their definitions and algorithms [7,8]. Despite disagreement over these connectivity models, researchers agree that involving species-specific perceptions of landscape determines, to a large extent, the success of a model in predicting potential connectivity between patches [7,9,10,11]. This consensus has motivated the concept of functional connectivity [7], which has rapidly expanded beyond stepping stones, migratory stopovers and habitat mosaics [2,10]. With the help of Graph Theory and Geographic Information System (GIS), a growing number of studies model least-cost functional connectivity between patches [12,13,14]. The functional model uses species-specific habitat preference, often based on expert opinion and literature [15,16], to depict the least-cost movement corridors or paths that provide better opportunities for successful dispersals. Advantages of this methodology include modest data requirements, a great benefit-to-effort ratio, visualized outputs and a convenient update process [7]. There exists, however, a common complaint about the expert-driven resistance estimates of landscape features, known as the “subjective translation problem” [17]. To improve the quality of and confidence in the least-cost algorithm, researchers suggest testing and refining the resultant map with empirical data, among others, results from landscape genetics are increasingly used [17,18,19].

**Figure 1:**
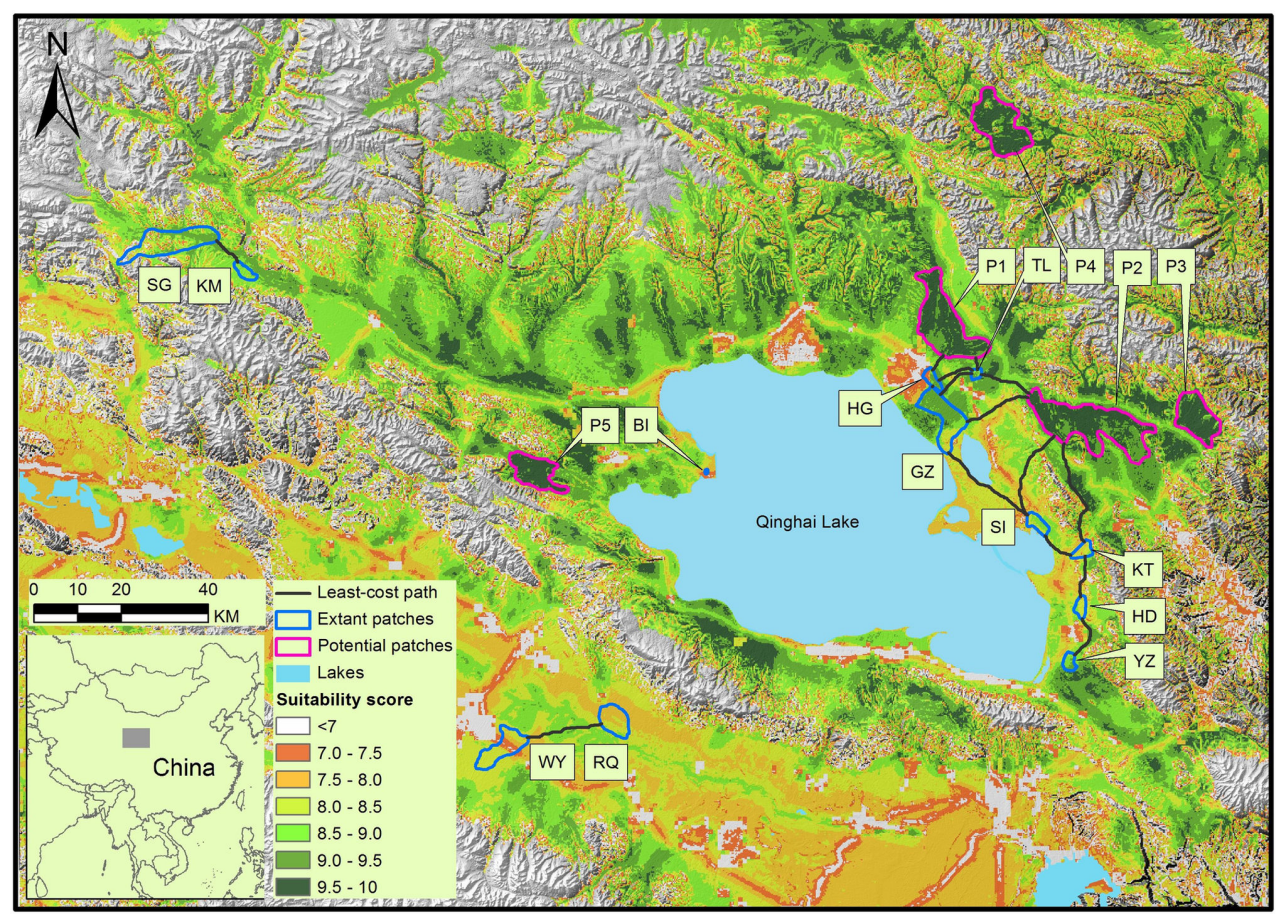
Habitat suitability map with extant and potential habitat patches of Przewalski’s gazelle. Przewalski’s gazelle are currently constrained in twelve isolated habitat remnants: Yuanzhe (YZ), Hudong (HD), Ketu (KT), Sand Island (SI), Ganzihe (GZ), Hargai (HG), Taliexuanguo (TL), Bird Island (BI), Kuairma (KM), Shengge (SG), Wayu (WY) and Ranquhu (RQ). The suitability scores range from one to ten, representing increasing habitat quality or movement resistance. Lakes in light blue and high mountains (> 4 000 m in elevation) in grey are avoided by the gazelle.

Przewalski’s gazelle, endemic to the Qinghai-Tibetan Plateau, is one of the most endangered antelope species in the world [20] and listed as Endangered on the IUCN Red List [21] and as a Category I Key National Protected Wild Animal Species in China [22]. The population underwent significant decline from the 1950s onwards and is now constrained to several isolated habitat remnants around the Qinghai Lake (Figure 1) [22,23,24]. Since the 1990s, local populations of Przewalski’s gazelle have experienced different trends [23,25]. Gazelle populations in patches experiencing either rebounds in population size or declines in habitat suitability may disperse to search for potential habitats [25,26]. On the other hand, Yang et al. [27] found significant genetic differentiation in the species, which call for gazelle dispersals between patches. Although habitats of the gazelle are relatively flat moraine covered with steppe, sand dunes and meadows, the gazelle may not move along the shortest Euclidean distance due to habitat degradation. In this context, it is necessary to assess functional connectivity among patches, including extant distributed areas and new potential habitats.

Thus far, except for a few inter-patch movements reported by local pastoralists and inferred by researchers [25,27,28], no studies have been conducted with regard to dispersal connectivity for Przewalski’s gazelle. Here, we present a spatially explicit model of habitat suitability and least-cost functional connectivity between the populations. We further evaluate and test the model using the latest evidence from landscape genetics of the gazelle [27]. This study presented thorough and visualized information for making conservation plans. In a general sense, we provided a heuristic framework to model landscape connectivity for species with similar biological characteristics and habitat patchiness to Przewalski’s gazelle.

## Methods

### Ethics Statement

Our research protocols were approved by the Chinese Wildlife Management Authority. The study is an ecological model with no cruelty to animals and no review from the ethnic committee is required in China. All work was conducted under the Wildlife Protection Law of the People's Republic of China.

### Modeling overview

We mapped habitat suitability and functional connectivity (least-cost corridors and paths) between habitat patches occupied by Przewalski’s gazelle based on Graph Theory, GIS and expert-literature method [10,18,29]. We first created a regional habitat suitability map and identified new potential habitat patches. Then we created a movement cost surface (permeability matrix) and modeled functional connectivity between neighboring pairwise habitat patches. We did not depict connectivity between patches over 25 km apart in that little or no gene flow of the gazelle had occurred beyond this threshold [27].

### Study area

Current distribution ranges of Przewalski’s gazelle are located around the Qinghai Lake (36°9’-37°56’N, 97°50’-101°6’E, Figure 1) [25]. The analytical window we used was large enough (58 610 km^2^) to include all the twelve extant distribution remnants, neighboring landscape and all potential looping corridors. The elevation of the analysis area ranges from 2 500 to 5 200 m. Dominant vegetation types include alpine meadow, alpine steppe, alpine shrub and psammophilous vegetation. The region has an inland plateau and typical semi-arid climate with dry, cold and long winters, a high level of solar radiation and a short frost-free period. Mean annual temperature is 0.5°C with the lowest record of -31°C and the annual precipitation is 350~420 mm. 

### Landscape features

After reviewing previous literature and consulting gazelle experts, we selected ten landscape features that were supposed to influence how Przewalski’s gazelle perceive landscape: elevation, slope, normalized difference vegetation index (NDVI), human density and distances to rivers, highways, railways, farmlands, settlements and individual houses. Raster layers of elevation, slope, NDVI and human density and vectors of river, highway and railway were downloaded from national websites (Table 1). The MODIS 16-day NDVI was composited by imageries from the 12^th^ to the 27^th^ of July 2010, the height of the growing season for plants on the grassland around the Qinghai Lake. Vectors of farmlands, settlements and individual houses were obtained with the help of high resolution (2.5m) Google^TM^ Earth imagery, which has been widely used in ecological and geographic studies [30,31]. Distances to the vectorial landscape features were calculated with the Spatial Analyst and saved in grid format. The ten raster layers were all projected in WGS 1984 UTM Zone 47N and rescaled to spatial resolution of both 250m and 30 m. All analyses were completed in ArcGIS 9.3 (ESRI 2008).

**Table 1:** Variables involved in mapping habitat suitability and functional connectivity for the endangered Przewalski’s gazelle.

Data base	Resolution	Year of data	Source
Elevation	30 m	2009	International Scientific Data Service Platform (http://datamirror.csdb.cn/)
Slope	30 m	2009	
NDVI	250 m	2010	
Human density	1 km	2003	Data Sharing Infrastructure of Earth System Science (http://wdcrre.geodata.cn/Portal/index.jsp)
River	1:4000000	2009	
Highway	1:4000000	2009	
Railway	1:4000000	2009	
Farmland	2.5 m	2008-2011	GPS locations collected during field surveys and Google^TM^ Earth
Settlement	2.5 m	2008-2011	
Individual house	2.5 m	2008-2011	

### Assigning suitability scores, movement cost scores and weights to landscape features

Before modeling habitat suitability and cost surface, cells in the raster layers were reclassified and each cell was assigned two integral scores (1-10) indicating habitat quality and movement resistance, respectively, for Przewalski’s gazelle. Due to the rarity of quantitative data, we employed the expert-opinion and literature review method [15]. If some cells are never selected as habitats or traversed by the gazelle, an N/A was assigned (Table 2 and 3). 

**Table 2:** Variables used in development of the habitat suitability model for Przewalski’s gazelle and expert-determined classification of landscape layers, corresponding suitability scores and weights.

Habitat suitability score*	Elevation (m)	Slope (degree)	NDVI	Human density (/km^2^)	Distance to (m)			
					river	highway	railway	farmland
1	< 3000	25-60	<0.1	>45	>4000	0-250		
2	3900-4000		0.1-0.2	40-45	3000-4000		0-250	0-250
3	3800-3900		0.2-0.3	35-40	2500-3000	250-500	250-500	
4	3000-3200	20-25	0.3-0.4	30-35				250-500
5	3700-3800		0.4-0.5	25-30	2000-2500	500-1000		
6	3600-3700	15-20	0.5-0.6	20-25	1500-2000			500-750
7	3500-3600		0.6-0.7	15-20		1000-1500	500-1000	
8	3400-3500	10-15	0.7-0.8	10-15	1000-1500			750-1000
9	3300-3400	5-10	0.8-0.9	5-10	500-1000	1500-2000		1000-1500
10	3200-3300	0-5	0.9-1.0	0-5	0-500	>2000	>1000	>1500
N/A†	>4000	>60						
Relative weights‡	0.0548	0.3891	0.1375	0.1813	0.0818	0.0964	0.0284	0.0307

* Suitability scores range from 1 to 10 representing low to high habitat quality. † An N/A value indicates that the gazelle never use the cells with those characteristics. ‡ Analytical Hierarchy Process (Saaty, 1987) was used to calculate the weights, which represent relative importance of variables to gazelle habitat selection.

**Table 3:** Variables used in development of the functional connectivity model for Przewalski’s gazelle and expert-determined classification of landscape layers, corresponding movement cost and weights.

Movement cost*	Elevation (m)	Slope (degree)	Distance to (m)				
			Highway	railway	farmland	settlement	individual house
10		25-60	0-90	0-30	0-90	200-300	
9	3900-4000	20-25	90-150				30-90
8		15-20		30-90	90-150	300-500	
7	3800-3900		150-200				90-150
6		10-15	200-300	90-150	150-200	500-1000	
5	3600-3800						
4			300-500	150-200	200-300	1000-1500	150-300
3	3400-3600	5-10	500-1000			1500-2000	
2			1000-2000		300-500	2000-2500	300-500
1	<3400	0-5	>2000	>200	>500	>2500	>500
N/A†	>4000	>60				<200	<30
Relative weights‡	0.0322	0.3875	0.1715	0.0477	0.0495	0.2095	0.1022

* Movement costs range from 1 to 10 representing low to high movement resistance. † An N/A value indicates that the gazelle never traverse the cells with those characteristics. ‡ Analytical Hierarchy Process (Saaty, 1987) was used to calculate the weights, which represent relative importance of variables to gazelle movement.

Relative influences of different landscape features in habitat selection and movement of Przewalski’s gazelle were indicated by weights that were calculated with AHP 1.1 (downloaded from http://arcscripts.esri.com/details.asp?dbid=13764) based on the Analytic Hierarchy Process (AHP) [32]. We asked experts to use a scale of 1-9 to represent the relative importance of one factor over another with 1 for equality and higher values indicating more prevalence. The procedure gave consistency ratios (CR) smaller than 0.1 (0.000 in habitat suitability model and 0.056 in cost surface model) indicating good consistency of answers between experts [32,33]. 

We did not perform sensitivity analysis in which the sensitivity of the model outputs were tested according to a range of suitability scores, movement cost scores or weights of landscape features [34]. We argue that this is reasonable given that the experts were all familiar with the species and we employed a more rigorous handling (AHP method) of the expert-based assignments. In addition, we asked experts to assign suitability scores and movement resistance separately because animals may not use the same rule to select habitats and movement paths [35].

### Modeling habitat suitability and identifying potential habitat patches

We modeled habitat suitability at 250 m resolution involving the effects of elevation, slope, NDVI, human density and distances to river, highway, railway and farmland. The suitability scores of cells in different layers were multiplied by the weights calculated with AHP 1.1. Then, Raster Calculator was used to combine the eight weighted landscape layers to generate a single habitat suitability map. Based on this map, we identified potential habitat patches using the Corridor Design Tool developed by Majka et al. [36]. We used relatively higher identification criteria (the patch size larger than 60 km^2^ and habitat suitability score higher than 9.50) with an aim to identify the potential patches of the high quality and priority. This is justified by the increase in the largest viable Ganzihe (GZ) population located in 2009 [25] with such patch size and suitability score. The boundaries of extant patches were delineated by connecting the peripheral GPS points of Przewalski’s gazelle during our 5-year field work.

### Modeling cost surface and functional connectivity at patch-to-patch level

Movement cost surface was modeled at 30 m resolution. Distances to settlements and individual houses were added as alternative proxies of human influence instead of human density which is inaccurate at fine scales [35]. This was done in addition to modeling habitat suitability (i.e., elevation, slope and distances to highway, railway and farmland). We did not either involve NDVI or distance to river because of their mixed effects on mobility of large herbivores. Cells with higher NDVI or rivers might attract gazelle and constrict their movements. A matrix with poorer primary productivity and longer distances to rivers, however, might unintentionally facilitate dispersal as gazelles need to search large areas for resources.

Raster layers of these seven features were combined with weights to generate a movement cost surface quantifying difficulty or ease for gazelles to traverse the landscape. With the cost surface, we used the tool, Cost Distance, to create cost distances for every patch indicating accumulated movement cost (shortest weighted distance) from each cell to the patch. Then the Corridor and Shortest Path functions were used to delineate least-cost patch-to-patch corridors and paths, respectively. The reclassified corridor map represented cumulative movement costs of embedded dispersal paths across the matrix between patches. The least-cost path was the single optimal route of travel with the least resistance. Previous studies extracted traversable corridor as slices of embedded paths of least movement cost. This method is arbitrary and untested in that the costs of the slices are not necessarily the threshold of dispersals. Another problem inherent in this extraction is that the procedure output is always a corridor, whether animals are able to travel or not [17]. Thus, it is impractical to set a realistic percentage threshold for dispersal. For these reasons, we compared connectivity between different pairwise patches using absolute accumulated movement cost.

## Results

Experts ranked slope as the strongest and elevation as the weakest in influencing both habitat selection and movement of Przewalski’s gazelle (Table 2 and 3). In the habitat suitability model, human density and NDVI followed the strongest factor. In the cost surface model, distance to settlement was the second strongest factor while distance to individual house was the fourth one. There were no disparities in ranking with the other landscape features, which had similar relative influencing weights between habitat suitability and cost surface. 

Among the extant distributed patches, average habitat suitability score was highest in SG (9.31) followed by GZ (9.25), HG (9.01), TL (9.01), YZ (8.80), KM (8.71), SI (8.60), HD (8.19), RQ (7.92), KT (7.76), WY (7.68) and BI (7.57). Patches with suitability higher than 9.50 covered a total area of 1 753 km^2^ (2.7% of the analytical window). Among these patches, five potential habitats (P1-P5) were identified and their area amounted to 638 km^2^ (1.1% of the analytical window). Of the five potential habitats, the shortest distance to current ranges was 2.5 km from TL to P1 and three existing patches (GZ, HG and TL) were in the vicinity of P1 (within 7.6 km). P2 was relatively further away but within 20 km distance from four populations (KT, SI, GZ and TL). P3, P4 and P5 were over 30 km away from proximate extant patches (33.1 km P3-KT, 49.0 km P4-TL and 31.6 km P5-BI) and isolated by high mountains or towns (Figure 1).

**Figure 2:**
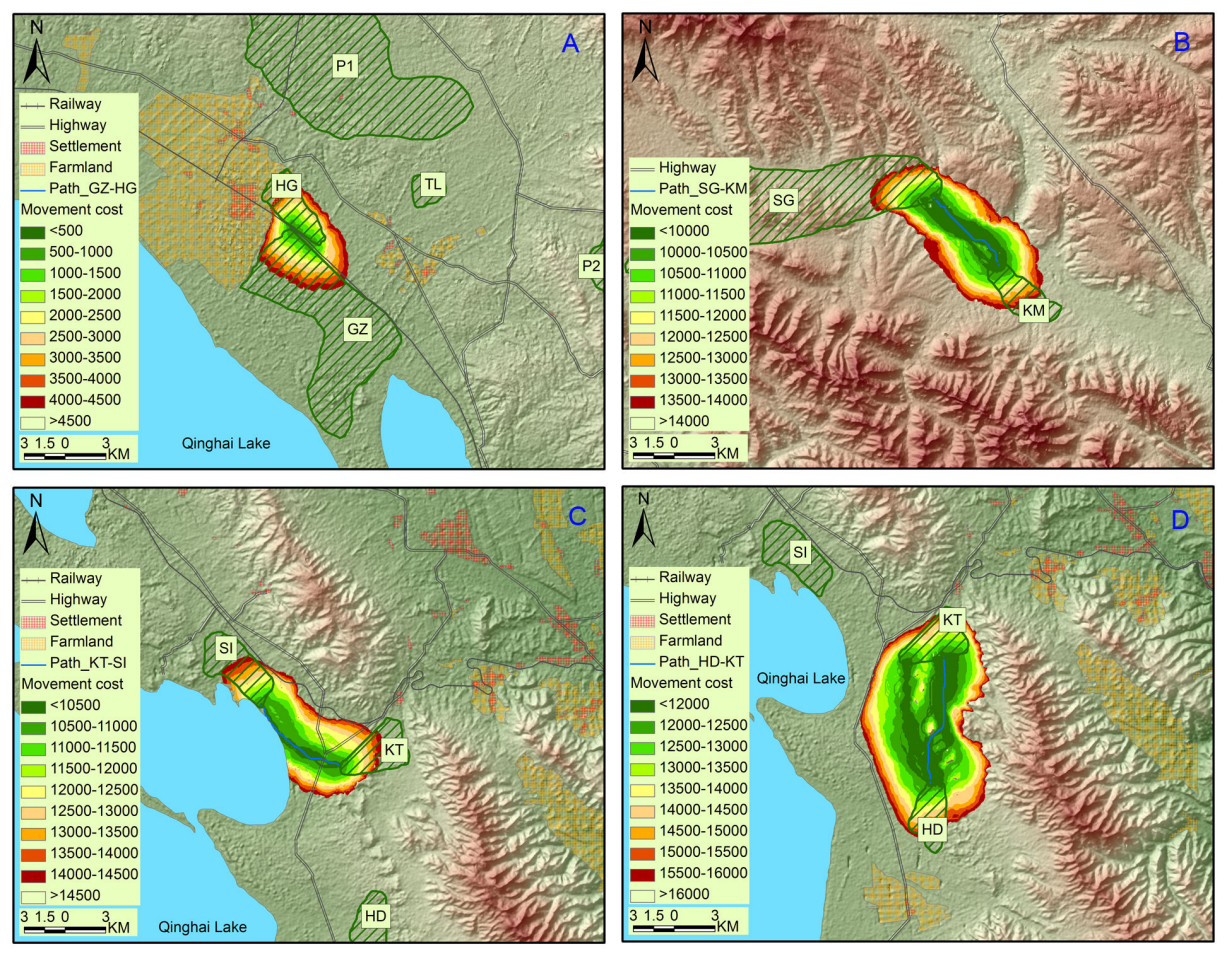
More traversable corridors and least-cost paths among extant populations of Przewalski’s gazelle. Four pairs of corridors and least-cost paths, GZ-HG (A), SG-KM (B), KT-SI (C) and HD-KT (D), were considered traversable if we set the cost value of the least-cost path HD-KT as a threshold for inter-patch dispersal.

**Figure 3:**
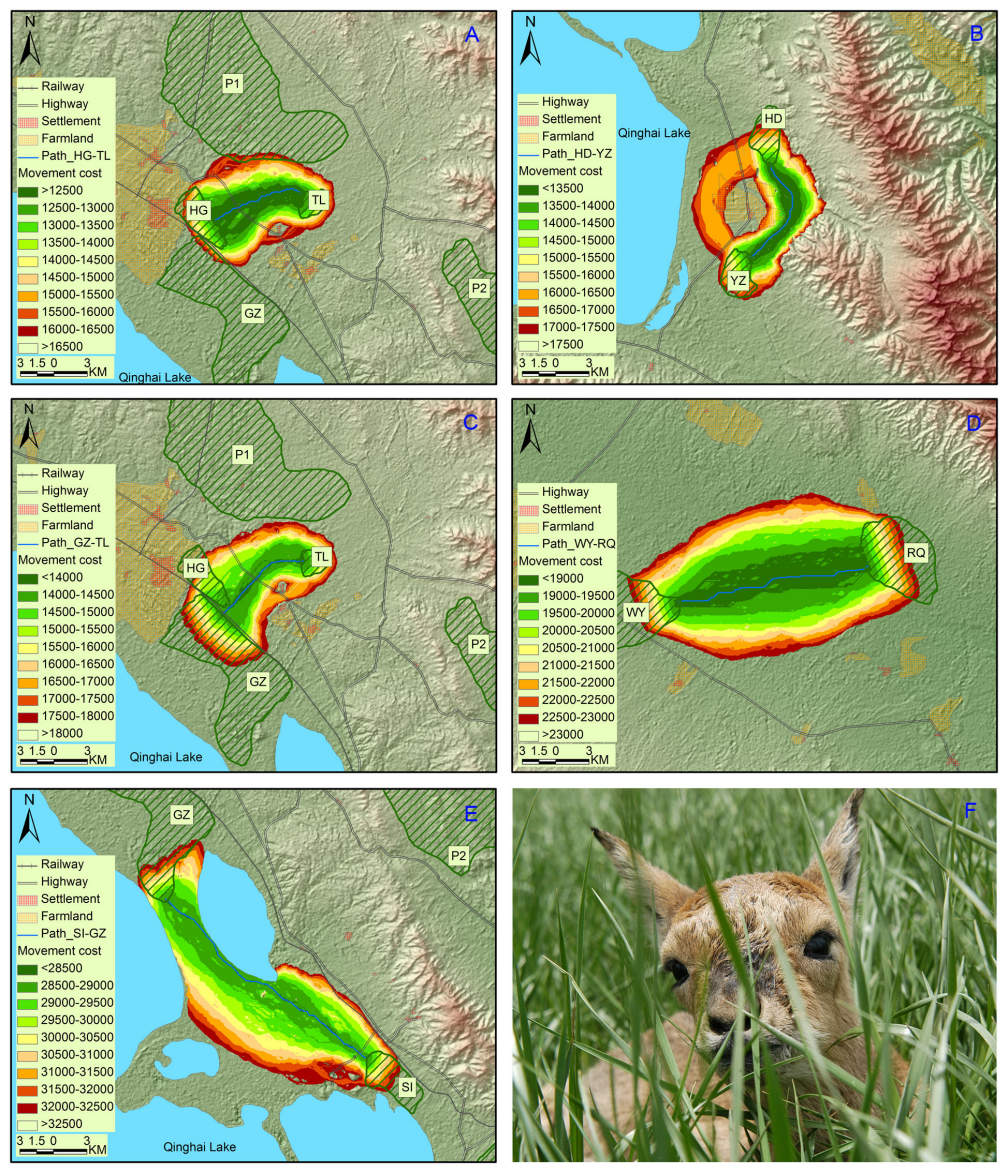
Less traversable corridors and least-cost paths among extant populations of Przewalski’s gazelle. Five pairs of corridors and least-cost paths, HG-TL (A), HD-YZ (B), GZ-TL (C), WY-RQ (D) and SI-GZ (E), were considered less traversable if we set the cost value of the least-cost path HD-KT as a threshold for inter-patch dispersal. A photo of the newborn Przewalski’s gazlle found in SG was displayed in panel F.

**Figure 4:**
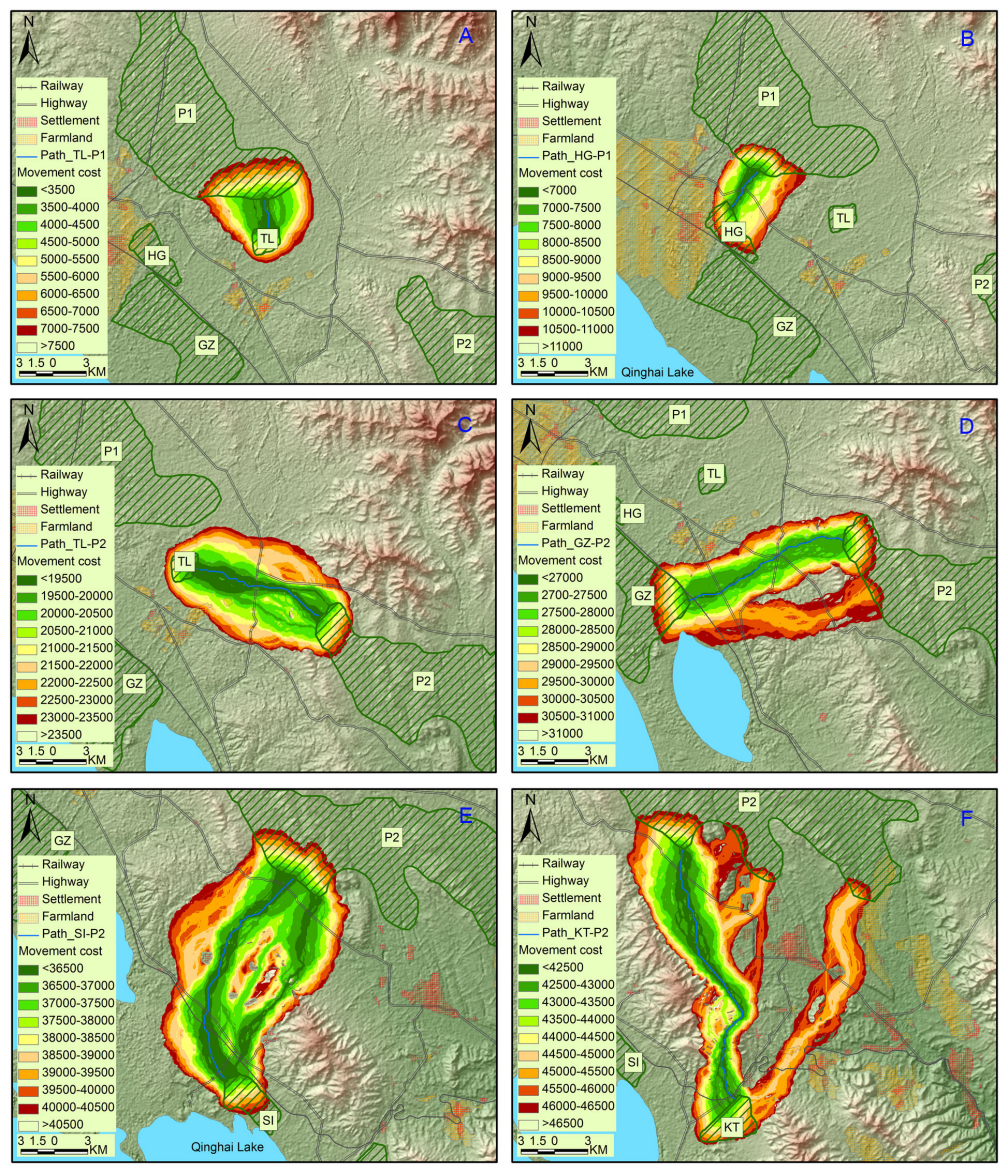
Corridors and least-cost paths between extant populations and potential patches of Przewalski’s gazelle. Six pairs of corridors and least-cost paths, TL-P1 (A), HG-P1 (B), TL-P2 (C), GZ-P2 (D), SI P2 (E) and KT-P2 (F), were mapped for Przewalski’s gazelle between potential patches and neighboring extant populations.

 Fifteen pairs of least-cost functional corridors and paths were depicted between neighboring pairwise patches including eleven extant and two potential patches (P1 and P2) within 25 km of one another (Figures 2, 3 and 4). Least-cost paths were all longer than the corresponding Euclidean distances except nearly equality between GZ and HG. Least-cost paths ranged from 0.2 km to 26.8 km (averaging 12.4 km) while Euclidean distances from 0.2 km to 22.8 km (averaging 10.2 km). Of the 15 least-cost paths, four held the cumulative movement cost lower than 10 000, four higher than 20 000 and seven in between. The least movement cost was found between GZ and HG (291) while the highest was between KT and P2 (42 268). The maximum cost of 1-km-corridor (corridor slice with minimum width of one km at the narrowest point along their length) ranged from 600 to 45 000, averaging 17 607. The 1-km-corridors of HD-KT, HG-TL, KT-P2, SI P2 and TL-P2 split into branches with several small areas of unsuitable landscape in the middle and near the edges. If we set the cost value of the least-cost path HD-KT (between which gazelle movements were considered present) as a threshold for inter-patch dispersal, there would be six traversable corridors, i.e., GZ-HG, TL-P1, HG-P1, SG-KM, KT-SI and HD-KT in descending ranking of permeability (Table 4).

**Table 4:** Summary of the 15 pairs of least-cost corridors and paths for Przewalski’s gazelle.

	Euclidean distance (km)	Least-cost path		Cost of 1-km-width corridor	Genetic differentiation (*F*'_*ST*_) †	Number of first-generation migrants†
		length (km)	movement cost			
GZ-HG	0.2	0.2	291	600	0.018 (± 0.024)	1
TL-P1	2.5	2.9	3198	3800	/	/
HG-P1	3.6	4.5	6778	7500	/	/
SG-KM	6.2	7.7	9541	10100	/	/
KT-SI	7.2	8.2	10398	11000	0.102 (± 0.122)	1
HD-KT	8.6	10.1	11558	12500	0.000 (± 0.008)	2
HG-TL	7.0	9.2	12039	12500	/	/
HD-YZ	8.2	10.9	13137	13700	0.115 (± 0.028)*	0
GZ-TL	7.6	9.6	13811	14400	/	/
WY-RQ	16.7	18.5	18733	19000	/	/
TL-P2	11.6	12.9	19145	20200	/	/
GZ-P2	14.6	16.7	26945	27500	/	/
SI-GZ	22.8	24.9	28468	29300	0.138 (± 0.072)	0
SI P2	17.4	23.1	36116	37000	/	/
KT-P2	19.4	26.8	42268	45000	/	/
Mean	10.24	12.4	16828	17607	/	/

† Standardized genetic differentiation (*F*’_*ST*_ with standard error) and number of first-generation migrants between patches examined by Yang et al. (2011) are also displayed for validation of the functional connectivity model. Significance level of *P*<0.05 is denoted with a “* ”. Those not examined by Yang et al. (2011) are shown as ‘/’.

## Discussion

We found that pastures in the study area were of low quality with limited suitable habitats for Przewalski’s gazelle in patchy landscape (Figure 1). Human expansion and excessive livestock grazing has caused deterioration of nearly all grasslands in the region [37]. Four populations (YZ, HD, KT and SI) at the eastern and BI population at the western shore of the Qinghai Lake are struggling to survive in restricted space neighboring to large sand dunes [25]. Two small populations (WY and RQ in 2008 and 2009, respectively) were newly found in degraded rangelands of the Gonghe Basin to the south of the Qinghainanshan Mountain. They might immigrate from the Qieji (QJ) population 20 km away driven down the Shazhuyu River by local degraded habitat [25]. In these areas, conservation efforts should be targeted at restoration of degraded habitats and human-gazelle conflicts [25,26]. Pastures of relatively higher quality for Przewalski’s gazelle are mainly identified in areas along the Buha River and those from the northern shore of the Qinghai Lake to the southern foot of Datong Mountain. This is consistent with the pattern predicted using the Maximum Entropy Approach [38,39], enhancing our confidence in the model output. 

 Our modeling indicated that regional and patch-to-patch connectivity for Przewalski’s gazelle is weak. Various barriers drive gazelles to face local extirpations due to stochastic events and inbreeding depressions [19,24]. On a regional scale, the barrier effects of natural features, including high mountains, lakes and long geographical distances, have existed for hundreds of thousands of years since the species formed [27]. According to these effects, Przewalski’s gazelle could be subdivided into four meta-groups: G1 (YZ, HD, KT, SI, GZ, HG and TL), G2 (BI), G3 (SG and KM) and G4 (WY and RQ). Movements between these meta-groups are few. In particular, the Qinghainanshan Mountain and a new highway along the southern foot of the mountain almost completely impede gazelle movement between Gonghe populations (G4) and those in the Qinghai Lake Basin (G1-G3). Genetic distances between Gonghe populations and the others (G1-G3) are the farthest among all pairwise populations [27]. In another study, evidence from skull morphology suggests that the genetic differentiation has reached the subspecies level in Gonghe populations [40]. 

Within each meta-group (G1-G4), anthropogenic barriers disable gazelle dispersals along Euclidean distances between patches. The settlement (Hudong) and surrounding farmlands bend the functional connectivity YZ-HD to the foot of a hill where human activities is less dense with scattered pastoralists’ houses. Similarly, connectivity SI-GZ is disrupted by a highway and a tourist attraction (Shaodao). Yang et al. [27] found significant genetic differentiation between these pairwise patches and local pastoralists reported no inter-patch migrants. National highway No. 315 with heavy car traffic runs between TL and the neighboring HG and GZ. The highway, together with the town Ganzihe, presents a barrier reducing gazelle movement. There is also a highway between KT and SI but with less traffic. The movement cost was relatively low and one first-generation migrant from KT to SI was detected [27]. This indicates a traversable connectivity between the two populations. However, the future of matrix permeability between the two remains uncertain due to increasing traffic commensurate with prosperous eco-tourism. The movement cost is the lowest between GZ and HG, which are located close to and separated by one fenced railway (Qinghai-Tibet). Local pastoralists reported gazelles passing through the eight underpasses (<10 m in width) and Yang et al. [27] suggest that the railway does not significantly impact the gene flow. The desert between HD and KT and streamside along Buha River between SG and KM are comparably traversable in that there are few barriers in between. Of the five identified potential patches, P1 has the shortest cost distance to the nearest extant populations (e.g., HG and TL) and P2 follows (in the vicinity of TL and GZ). The connectivity WY-RQ is relatively straight due to the homogeneity of the landscape across the area. The degraded grassland in the basin may unintentionally facilitate movement between the two patches.

The reduced landscape connectivity for Przewalski’s gazelle could be enhanced in case of implementing appropriate restoration programs. Habitat restoration inside and between patches should be a priority [13,25,38]. Overgrazing should be controlled by reducing the size of domestic livestock. Human activities inside or near the 1-km-corridors, including settlements, tourism attractions and livestock herding, should be controlled to a lesser degree. Following habitat restoration, inter-patch corridors could be restored wide enough that they could be more resilient to edge effects, climatic changes and anthropogenic influences [2,17]. Barrier effect of transportation routes could be mitigated by construction of highway crossing structures, which have been applied successfully in many other species [41,42,43,44]. However, there are currently no under- or over-crossing structures on the highways in the Qinghai Lake region. The existing railway underpasses are either not specially designed for wild ungulates. An immediate recommendation is to construct wider crossing structures where least-cost corridors intersect with transportation routes. Additionally, the conservation of northern populations (HG, GZ and TL) should be prioritized in the sense of preserving a viable source of the gazelle metapopulation. These populations have nearly half of the gazelles (736/1544=47.7%), and the populations are increasing [25]. If the connectivity from these populations is preserved or regenerated, they will be beneficial in maintaining genetic diversity, sustaining extant metapopulation and occupying new potential habitats to the north. 

There exists a common controversy over the ‘subjective translation’ of the expert-opinion and literature method [17]. However, this method has been used in many connectivity studies for a wide range of vertebrates and invertebrates [17,45] and found to be a good fit to independent empirical data [13,15,16]. The knowledge about Przewalski’s gazelle of the experts we invited in this study help improves the model. Another limitation is that we did not include the effect of grassland fencing which does harms to gazelles [25,46,47]. It was impractical to locate or model the spatial configuration of the fencing at a regional scale. However, we argue that the exclusion has little effect on the model outputs because grassland fencing is distributed homogeneously inside and between patches.

The functional connectivity modeled here is really a starting point rather than a final solution. It is a putative model based on expert opinions and literature reviews rather than depicted using large frequently sampled dataset of locations obtained by GPS or radio telemetry [1,12]. We concur that Przewalski’s gazelle does not necessarily use the least-cost corridors given the complex and stochastic attributes of animal movement [2,4]. The modeling, however, identified a swath of lands with higher permeability, providing the most potential opportunities for successful dispersals between patches. Findings in landscape genetics [27], our previous field work and allegations by local pastoralists have provided first-step validation of the connectivity pattern. Before corridors are finalized, model outputs should be further assessed by field surveys of actual use of corridors by Przewalski’s gazelle. Following this, we encourage studies on gazelle movement with the help of GPS telemetry.

As Mallon and Jiang [48] pointed out: conservation of wild grazing ungulates in Central Asia in the long run presents a challenge, especially when considering migratory behavior of the species in nomadic landscape. However, conservation biology addresses crises in biodiversity preservation [49] and we should take immediate actions when there remain populations of Przewalski’s gazelle and some suitable habitat remnants. Such conservation impetus may also be provided by the fact that the gazelle still has an evolutionary potential and moderate nuclear genetic diversity in spite of a genetic bottleneck [27,28]. The habitat suitability and functional connectivity modeled in this study provides conservation practitioners with an initial, but thorough and visualized, look at the connectivity network for Przewalski’s gazelle. In conjunction with previous research findings on the gazelle [25,26,27,38,39,50,51], this study has direct applications to decision making process in which limited funding could be targeted at those patches and corridors identified as conservation priorities. In a general sense, we suggest that decision makers should involve habitat connectivity in conservation planning for species with similar biological and ecological characteristics living in patchy landscape.
