# Correction: A Spatially Explicit Model of Functional Connectivity for the Endangered Przewalski’s Gazelle (*Procapra przewalskii*) in a Patchy Landscape

**DOI:** 10.1371/annotation/8cf2e747-87cb-4415-87ae-d643f63b6288

**Published:** 2013-12-10

**Authors:** Chunlin Li, Zhigang Jiang, Hongxia Fang, Chunwang Li

The number of the grant from the Natural Sciences Foundation of Education Department of Anhui Province is incorrect. The correct grant number is: KJ2013A023.

